# Impact of network performance on remote robotic-assisted endovascular interventions in porcine model

**DOI:** 10.1007/s11701-021-01196-6

**Published:** 2021-02-07

**Authors:** Peter Legeza, Gavin W. Britz, Alpesh Shah, Kalyna Sconzert, John-Michael Sungur, Ponraj Chinnadurai, Kavya Sinha, Alan B. Lumsden

**Affiliations:** 1 Department of Cardiovascular Surgery, Houston Methodist Hospital, 6550 Fannin St, Suite 1401, Houston, TX 77030 USA; 2 Department of Vascular Surgery, Semmelweis University, Budapest, Hungary; 3 Department of Neurological Surgery and Neurological Institute, Houston Methodist Hospital, Houston, USA; 4 Houston Methodist DeBakey Cardiology Associates, Houston Methodist Hospital, Houston, TX USA; 5 Corindus, A Siemens Healthineers Company, Waltham, MA USA; 6 Siemens Medical Solutions USA Inc., Hoffman Estates, Illinois, USA

**Keywords:** Robotic-assisted, Endovascular surgery, Remote surgery, Network latency, Robotic-PCI

## Abstract

Remote robotic-assisted endovascular interventions require real-time control of the robotic system to conduct precise device navigation. The delay (latency) between the input command and the catheter response can be affected by factors such as network speed and distance. This study evaluated the effect of network latency on robotic-assisted endovascular navigation in three vascular beds using in-vivo experimental model. Three operators performed femoral, carotid, and coronary endovascular robotic navigation blinded from the hybrid room with the prototype remote-enabled CorPath GRX system in a porcine model. Navigation was performed to different targets with randomly assigned network latencies from 0 to 1000 ms. Outcome measurements included navigation success, navigation time, perceived lag (1 = imperceptible, 5 = too long), and procedural impact scored by the operators (1 = no impact, 5 = unacceptable). Robotic-assisted remote endovascular navigation was successful in all 65 cases (9 femoral, 38 external carotid, 18 coronary). Guidewire times were not significantly different across the simulated network latency times. Compared to 0 ms added latency, both the procedural impact and perceived lag scores were significantly higher when the added latency was 400 ms or greater (< 0.01). Remote endovascular intervention was feasible in all studied anatomic regions. Network latency of 400 ms or above is perceptible, although acceptable to operators, which suggests that remote robotic-assisted femoral, carotid or coronary arterial interventions should be performed with network latency below 400 ms to provide seamless remote device control.

## Introduction

Robotic-assisted endovascular interventions have multiple advantages compared to conventional, manually performed interventions. They play a significant role in reducing occupational hazards for the operator, such as radiation exposure and orthopedic complications due to heavy lead aprons [1, 2]. With the use of telecommunication, robotic-assisted interventions can be performed from great geographical distances. This method could increase access to care for patients with limited access to appropriate health services, due to geographic barriers, or could allow remote proctoring for certain procedures.

The feasibility of percutaneous coronary intervention (PCI) from long geographic distances has been previously described in both animal models and humans [3–5], but tele-robotic peripheral and neurovascular intervention has not been demonstrated yet.

During remote robotic-assisted navigation, the operators use joysticks to manipulate the endovascular devices. Real-time response of the system is required to carry out precise navigation between the remote and local sites, which highly depends on network performance. The effect of network latency during coronary artery navigation and PCI has been demonstrated, but no data is available on its effect, when robotically navigating in the peripheral arterial system. This preclinical study aims to evaluate the effect of network latency on robotic-assisted endovascular navigation in coronary, lower extremity and extracranial arteries using an in vivo experimental model.

## Materials and methods

### The robotic system

The CorPath GRX (Corindus, A Siemens Healthineers Company, Waltham, Massachusetts) is a robotic system, FDA-cleared for coronary and peripheral vascular interventions and CE-marked for neurovascular interventions. The robot allows the operator to deliver and navigate guidewires, rapid exchange devices and guide catheters via joystick control. The system is compatible with commercially available 0.014-inch guidewires and standard rapid exchange (RX) balloons and stent delivery systems. The bedside component includes a single-use sterile cassette, which advances, retracts, and rotates the guidewire and the guide catheter, advances and retracts the RX device. This cassette is attached to a robotic drive that can advance and retract the guide catheter with its forward and backward movements. With the use of the advanced cassette, the system is also capable of manipulating microcatheters. The robotic drive is supported by the extended reach arm, which is mounted on the bedside rail.

The operator sits in a radiation-shielded interventional cockpit and navigates the devices via the control console. The control console consists of a touchscreen and three joysticks for RX device, guidewire and guide catheter manipulation.

**Fig. 1:**
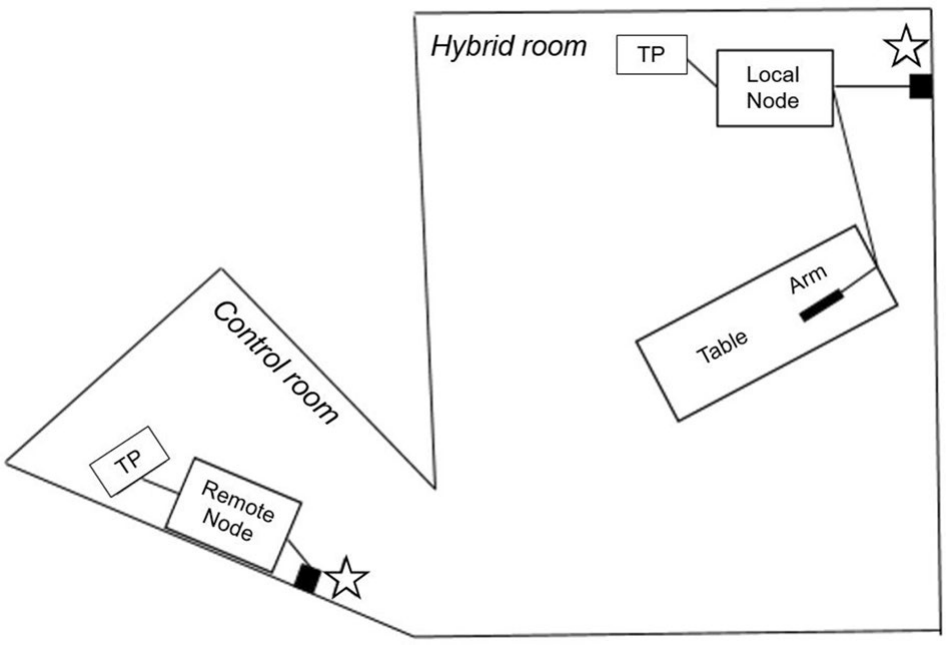
Layout of the hybrid suite and the remote workstation. The operator performed the robotic navigation from the control room, facing away from the hybrid room. Connection was achieved between the two nodes via institutional network connection (stars). *TP* telepresence system, *Table* operating table, *Arm* robotic arm

The present study utilized a remote prototype modification of the CorPath GRX system, which allowed the physical separation of the remote and local nodes, while connected via the institutional network. The robotic control unit and drive were connected to a target computer (Mobile RT, Speedgoat, Inc., Natick, MA) that each utilized a grandmaster clock and global positioning system antenna for synchronization of the control unit with the robotic drive. In this study the in vivo model, robotic arm, and bedside technician were located in a hybrid interventional suite (“local”), while the operators were navigating the endovascular interventional devices from the control room (“remote”), facing away from the hybrid room (Fig. 1).

The remote workstation includes monitors that display angiographic images, reference images, and hemodynamic data, similar to a typical CorPath GRX case. A telepresence system (LifeSize, Austin, TX) was used to communicate via live audiovisual streams of the operating room, operational field, and remote workstation.

Network latency was simulated by delaying robotic commands in the remote prototype modification of the CorPath GRX System. Simulated latencies ranged from 0 to 1,000 ms and were additive relative to the low (but nonzero) native latency of the institutional network.

### Procedural details

The procedure was performed in a domestic cross, female swine weighing 49.1 kg. The animal procedure was conducted under a protocol approved by the Institutional Animal Care and Use Committee.

The animal underwent standard anesthesia procedure by a clinical veterinarian and the access site was prepared and draped. Right common femoral access was gained, and a 6 French sheath (Destination; Terumo Interventional Systems, Somerset, NJ) was placed in the contralateral common femoral artery under fluoroscopy guidance (Zeego; Siemens Healthineers, Malvern, PA). Femoral arterial robotic navigation was performed with a 0.014″ (BMW; Abbott Vascular, Santa Clara, CA) or 0.018″ wire (V18; Boston Scientific, Marlborough, MA) and a 0.027″ microcatheter (Renegade; Boston Scientific, Marlborough, MA). For the external carotid arterial approach, a 7F Envoy guide catheter (90 cm) (Codman, Raynham, MA) was placed in the ostium of the left common carotid artery with a 0.014″ wire (Synchro2, Stryker Neurovascular, Fremont, CA) and a Renegade Hi-Flo microcatheter (0.027″ ID, 135 cm). For coronary arterial navigation, a Q3.5 guide catheter (Boston Scientific, Marlborough, MA) was placed in the ostium of the left coronary artery with a 0.014″ wire.

Angiography was performed in each vascular bed after the sheath was placed in the ostium of the target vessel, and based on the angiography, navigational targets were defined. These targets were a distal deep femoral branch, lingual artery, branch of the facial artery, and the diagonal branch of the left anterior descending coronary artery.

### Navigational tasks

A vascular surgeon, a neurosurgeon, and an interventional cardiologist participated, and performed femoral, external carotid and coronary arterial navigation, respectively. Each operator has completed > 20 endovascular robotic cases in both ex-vivo and in-vivo cases. Their task was to robotically navigate the guidewire from the tip of the guide catheter to the preselected targets. After the navigation to a target was completed, the wire was retrieved in the tip of the guide catheter, and the run was repeated.

During different runs, randomly assigned robotic command delays (latencies) were added to the system ranging from 0 to 1000 ms (0, 150, 250, 400, 600, 1000 ms). LAN connection includes an intrinsic command and image delay, which was incremental to the injected latency times. Operators were blinded to latency times. The first four runs were performed with 0 ms added latency, then random latencies were added to each run, up to 19 runs total per navigational target.

After finishing the endovascular navigational tasks, devices were removed, the access site was closed, and the animal was euthanized.

### Data analysis

The success of guidewire navigation was defined as the ability to navigate the guidewire from the tip of the guide catheter to the preselected target. For each run, guidewire times were measured. After every run, operators scored the perceived latency and the procedural impact on a scale from 1 to 5. Perceived latency was scored by the operators according to the following scale: 1 = imperceptible; 2 = noticeable but minor; 3 = noticeable; 4 = noticeable and major; 5 = too long. Procedural impact was scored by the interventionalist according to the following scale: 1 = no impact; 2 = minor impact (acceptable performance); 3 = noticeable impact (loss in efficiency, successful outcome); 4 = significant degradation (can complete, but not desired); 5 = unacceptable to complete. Data were analyzed across the three vascular beds, and also separately by making two groups: peripheral (femoral and external carotid) and coronary.

Categorical data are presented as count (*n*) and percentage (%), continuous variables are summarized as mean and standard deviation (SD). A Kruskal–Wallis test was conducted to determine if wiring times, perceived latency scores and procedural impact scores were different for among the injected latencies. *P* for trend was obtained from a Wilcoxon-type test for trend to evaluate the trend in perceived latency and procedural impact scores. Results were considered significant when *p*-value was < 0.05. Post-hoc tests for comparing added latency of 0 ms with added latencies up to 1000 ms were performed with the Man-Whitney test. STATA statistical software (StataCorp LP, College Station, TX, USA) was used for statistical analysis.

## Results

### Procedural success and guidewire navigation time

All 65 robotic-assisted guidewire navigation attempts with the added latencies from 0 to 1000 ms were successful (9 femoral, 13.8%; 38 external carotid, 58.5%; and 18 coronary, 27.7%). Mean wire navigation time for femoral, carotid target no. 1, carotid target no. 2 and coronary target were 131 ± 84.25, 26.26 ± 29.66, 104.9 ± 84.25 and 70.22 ± 65.18 s, respectively. Across the range of latencies studied, there was no significant difference and no trend observed in navigational times within each vascular bed.

### Procedural impact and perceived latency scores

**Fig. 2:**
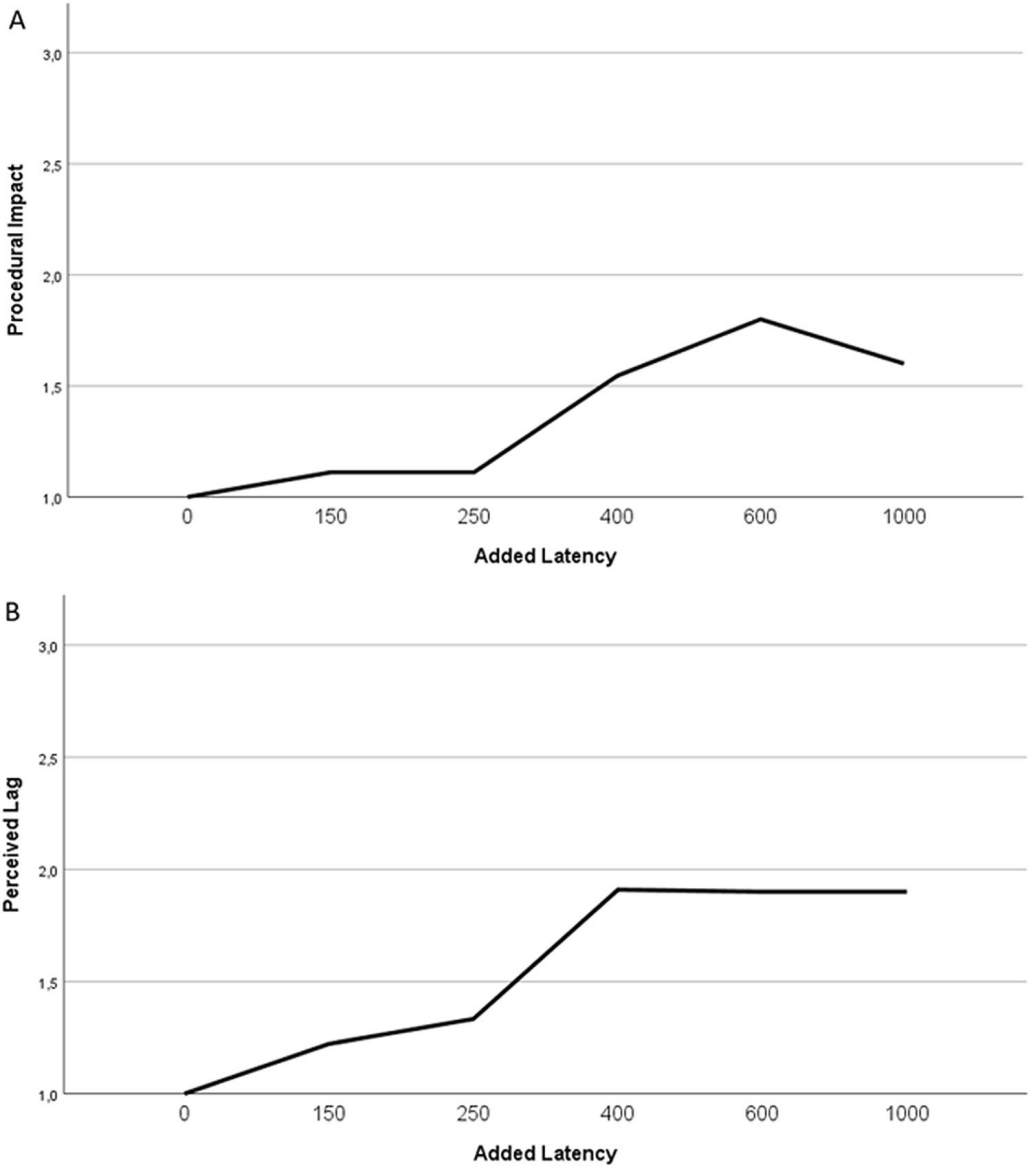
**a** Overall procedural impact score (mean ± SD) with different added command latencies (ms), **b** Overall perceived latency score (mean ± SD) with different added command latencies (ms)

**Fig. 3:**
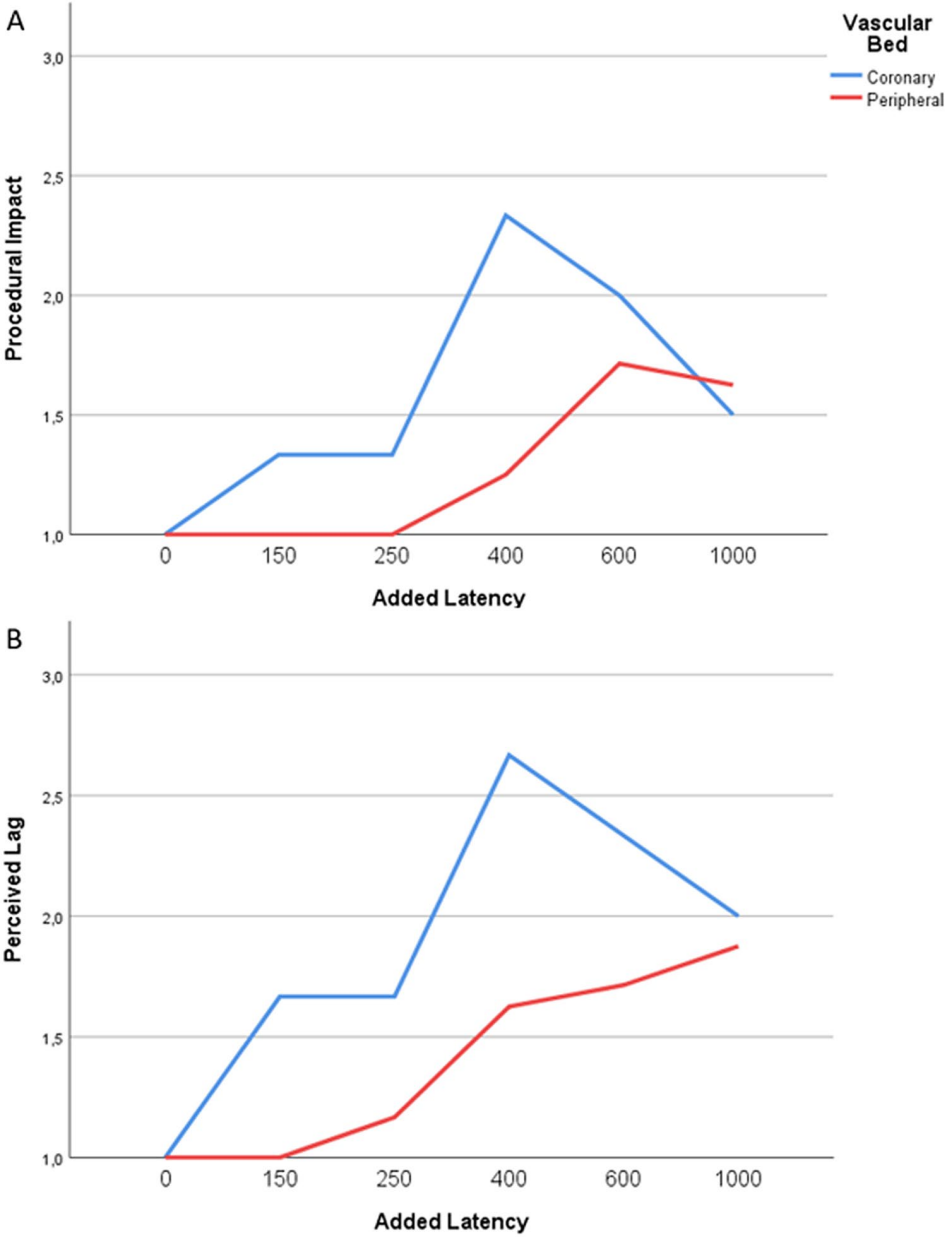
(**a**) Procedural impact score (mean ± SD) with different added command latencies (ms), **b** Perceived latency score (mean ± SD) with different added command latencies (ms). Blue: coronary arterial navigation, red: peripheral arterial navigation

There was a significant trend for higher procedural impact and perceived latency scores across the three vascular beds with the increasing latencies (*p* = 0.006 and *p* = 0.002, respectively) (Fig. 2). Across the three vascular beds, a significant difference was shown in the distribution of procedural impact (*p* = 0.048) and the perceived latency (*p* = 0.038) scores. When analyzing the scores between the peripheral and the coronary navigation, a tendency of higher scores was seen with the longer added latencies but the difference was not significant (Fig. 3).

For both the procedural impact and perceived lag scores, post-hoc analysis showed no significant differences when comparing the scores between the baseline latency of 0 ms to 150 and 250 ms, but the scores were significantly different when the added latency was 400 ms or greater (*p* < 0.05)(Tables 1, 2).

**Table 1:** Mean procedural impact scores with different added command latencies

Added latency (ms)	Procedural impact score	*p*-value
0	1	N/A
150	1.07 ± 0.7	0.548
250	1.11 ± 0.33	0.55
400	1.55 ± 0.93	0.03
600	1.8 ± 1.23	0.01
1000	1.67 ± 1	0.02

The scores with added latencies were compared to scores with 0 ms added latency

**Table 2:** Mean perceived latency scores with different added command latencies

Added latency (ms)	Perceived latency score	*p*-value
0	1	N/A
150	1.14 ± 0.1	0.32
250	1.33 ± 0.5	0.13
400	1.91 ± 1.04	< 0.01
600	1.9 ± 1.45	0.03
1000	2 ± 1.41	0.02

The scores with added latencies were compared to scores with 0 ms added latency

## Discussion

Remote robotic assistance in endovascular surgery allows the operator to perform the intervention even while the operator is physically in a separate location from the patient. Although locally performed robotic-assisted endovascular procedures have been introduced to interventional cardiology, peripheral, and neurovascular surgery [1, 6–8], previous tele-robotic studies only investigate the feasibility and characteristics of percutaneous coronary interventions [3, 5].

The operating team has to overcome several challenges when performing remote endovascular interventions, including the quality of network connection. Previous studies have shown that high-quality network connection is required for the safe navigation of the surgical devices in real-time, and to provide stable audiovisual connection between the local and remote site. A tele-robotic surgical study determined an acceptable limit of 330 ms time delay [9]. Madder et al. came to the conclusion that latencies during tele-robotic coronary navigation were perceptible for the operator at 400 ms or above [3].

This study investigated the feasibility of remote, robotic-assisted endovascular navigation in femoral, external carotid, and coronary arteries, and assessed the effect of network performance on the success of endovascular navigation. The setting of the study simulated an environment of remote intervention with operators who were connected to the hybrid room through a telepresence system.

Since the operator’s tasks only involved navigation, the arteries selected required complex wire and catheter manipulation by the operator. The 100% success rate of navigation to these preselected targets, even with the added command latencies, is a promising result in terms of precise robotic control. Our current knowledge of robotic-assisted peripheral vascular interventions is based on simple anatomic configurations [7]; however, the successful navigation to all targets in this study suggests that future applications of the robot may include peripheral cases, where complex navigation is needed.

Guidewire navigation time did not show a significant difference when analyzed across the different latency times and different anatomical regions, which may be due to the physician’s ability to compensate for a delay of up to 1000 ms. No data were collected on latencies above 1000 ms, thus we do not know if latencies outside of the tested range influence the guidewire navigational times or not.

Compared to 0 ms added latency, a significant increase in the procedural impact scores at 400, 600 and 1000 ms latency were shown. At these latency values the operators did experience the impact of the network latency during navigation. However, this increase in the scores only indicated a minor effect on their performance of the procedure (Table 1). The perceived latency scores were significantly higher at 400 ms and above. Based on the scores given by the operators, the perception of the latency at these latency values was “noticeable but minor” (Table 2).

When estimating the threshold of the network latency for remote robotic control, even minor noticeable changes in the network quality should be considered. According to the findings of this study the suggested value of seamless robotic control should be below 400 ms.

It is important to outline that our results are only showing the effect of network performance on navigation, and not ballooning or stent deployment. These procedures may require even more harmony between the local and remote sites, so acceptable network latency values may be lower during these maneuvers. Further studies are needed to evaluate the impact of network performance on robotic-assisted interventional procedures.

The limitations of this study are the single porcine model and the repetitions of the same tasks multiple consecutive times. This study only evaluated wire, microcatheter, and guide catheter navigation, but no balloon angioplasty or stenting was performed. The number of navigational runs per targets were too low; therefore, the statistical power was not high enough to see significant results, when analyzing the targets separately. Besides the guidewire navigation time, no other objective measurements were made, including fluoroscopy time and radiation doses.

This study is an important first step in understanding the parameters which are influencing the performance of remote endovascular interventions.

## Conclusion

Robotic-assisted femoral, external carotid and coronary navigation are feasible under remote conditions. No influence was demonstrated on the time of guidewire navigation to the target across the range of tested latencies (0–1000 ms). Operators reported a minor impact on their performance with latencies of 400 ms or above. Network latency of 400 ms and above was reported to be perceptible but acceptable. These results suggests that remote robotic-assisted femoral, carotid, or coronary arterial interventions should be performed with network latency below 400 ms to provide seamless remote device control.

## Data availibility

Available upon request.
